# *‘We are experts in telling our story’*: the perspectives of stakeholders from Aboriginal Community-Controlled Health Services on the health and wellbeing of urban First Nations Australians, and their priorities for a First Nations urban health research agenda in Australia

**DOI:** 10.1017/S1463423625100807

**Published:** 2026-01-28

**Authors:** Anton Clifford-Motopi, Janet Stajic, James Ward, Anthony Shakeshaft

**Affiliations:** Poche Centre for Indigenous Health, The University of Queensland, Brisbane, Australia

**Keywords:** health and wellbeing, primary health care, research priorities, urban First Nation Australians

## Abstract

**Background:**

The urban First Nations population in Australia is rapidly increasing. The health policy and research focus on urban First Nations Australians, however, is limited. To contribute to addressing this situation, The University of Queensland Poche Centre for Indigenous Health (UQ Poche Centre), a First Nations-led health research centre, is working closely with urban Aboriginal Community-Controlled Health Services (ACCHS) across Australia.

**Aim:**

Our study examined urban ACCHSs stakeholders’ perspectives of the health and wellbeing of urban First Nations Australians and identified their priorities for a national Indigenous urban health research agenda.

**Methods:**

Ten stakeholders were recruited for in-depth interviews from ACCHS that were members of the Research Alliance for Urban Community-Controlled Health Services (RAUCCHS), a partnership between the UQ Poche Centre and urban ACCHS focused on achieving equitable health outcomes for urban First Nations Australians. Six stakeholders identified as First Nations Australians. Interviews were audio-recorded and transcribed verbatim. Interview data were analysed using inductive thematic analysis.

**Results:**

Stakeholders highlighted a lack of research focused on the health of urban First Nations Australians. Specific priority areas they identified for an urban First Nations health research agenda were: evaluating the effectiveness and adaptability of Indigenous models of care, strengthening care pathways between ACCHS and specialist services, examining the intersection of cultural identity, racism and determinants of health, and greater investment in Indigenous research governance structures and processes.

**Conclusions:**

There is a clear opportunity for researchers to engage with RAUCCHS members to establish a body of urban First Nations health research in Australia that responds to their research priorities.

## Introduction

Between 2011 and 2021, the population of Aboriginal and Torres Strait Islander peoples (hereon referred to as First Nations Australians) residing in capital cities increased by 67%. At the same time, the population of non-Indigenous Australians residing in cities increased by 21% (Australian Bureau of Statistics, 2022). Despite the rapid increase in urban First Nations populations in Australia, there is limited policy and research focus on their health and wellbeing. And yet, in 2018, First Nations Australians residing in urban areas accounted for 56% of the total disease burden for all First Nations Australians. (Australian Bureau of Statistics, 2022). Several key factors play into this including: an increasingly urbanized population in Australia; a much younger demographic and rapidly growing First Nations Australian population compared to the general Australian population (Australian Bureau of Statistics, 2022); a lack of data especially health and wellbeing data disaggregated to each capital city; and a common discourse in First Nations health to focus on remote/regional areas, based on an incorrect, underlying assumption that urban First Nations peoples can access major mainstream tertiary and specialist services close to where they live (Mamun *et al.,* 2023). Adequately responding to these factors is essential to improve life expectancy (National Indigenous Australians Agency, 2021a), health outcomes (National Indigenous Australians Agency, 2021b; Australian Institute of Health and Welfare, 2021b), and health system performance for First Nations Australians (Australian Institute of Health and Welfare, 2021a).

There is evidence that adequately resourced and implemented First Nations’ health care in cities can result in significant improvements in health care access, utilization, and outcomes for First Nations Australians (Driscoll *et al.,* 2013; Kildea *et al.,* 2019). Despite this, reviews of First Nations’ health research continue to highlight major gaps in research effort and investment targeting First Nations Australians in urban areas. In 2010, a review, led by Eades (Eades *et al.,* 2010) found that just 11% of all articles in the previous five years focused on urban Indigenous health, despite almost 55% of the total Indigenous Australian population living in urban areas (including inner regional areas). In 2021, Jennings et al., published a rapid review showing that up to 3 times as many published research papers focused on remote Indigenous health than urban Indigenous health (Jennings *et al.,* 2021). Similarly, a narrative review of Indigenous health publications from 2008 to 2020, reported that studies were most commonly conducted in remote settings (Kennedy *et al.,* 2022). In order to ameliorate First Nations’ health disadvantage nationally, more research investment and effort on urban populations and communities is required (Stajic *et al.,* 2023).

In 2021, The University of Queensland’s (UQ) Poche Centre for Indigenous Health established partnerships with urban Aboriginal and Torres Strait Islander Community-Controlled Health Services (ACCHS) to form the Research Alliance for Urban Community-Controlled Health Services (RAUCCHS) (The UQ Poche Centre for Indigenous Health, 2023). As an enactment of self-determination, Aboriginal and Torres Strait Islander Community-Controlled Health Services (ACCHS) have led the way in innovative service delivery through the provision of culturally responsive holistic primary health care for First Nations Australians (Panaretto ; Turner *et al.,* 2019; Pearson *et al.,* 2020). The overall purpose of RAUCCHS is to bring together and utilize the expertise and capacity of urban ACCHS to achieve equitable health outcomes for urban First Nations Australians through transformational changes in health systems, policy and care (Stajic *et al.,* 2023; The UQ Poche Centre for Indigenous Health, 2023). In this paper, we report on qualitative interviews with stakeholders from urban ACCHS that are members of RAUCCHS. The purpose of interviews was to gather information from ACCHS stakeholders to contribute to the development of the *UQ Poche Centre Indigenous Urban Health Research Agenda*, to ensure that research conducted by UQ Poche aligns with the research priorities of urban ACCHS. This paper examines urban ACCHS stakeholders’ perspectives of the health and wellbeing of urban First Nations Australians and identifies their priorities for a national Indigenous Urban Health Research Agenda.

## Methods

### Ethics

Ethical clearance was obtained from the University of Queensland Human Research Ethics Committee (ID number: 2022/HE001932). All participants provided informed written consent prior to their participation in the study.

### Methodological approach

Our study was a qualitative design using semi-structured individual interviews. The study was conceptualized by a senior First Nations Australian researcher responsible for leading the development and implementation of the research agenda at the UQ Poche Centre for Indigenous Health. The researcher developed the interview questions and had oversight of the implementation of study methodology. The Consolidated Criteria for Reporting Qualitative Research (COREQ) checklist was used to guide the implementation and reporting of study methodology (Tong *et al.,* 2007) (File S1).

### Participants

We purposively recruited stakeholders from the eleven urban ACCHS that were members of RAUCCHS at the time of the study. As the overall goal of the study was to gain a rich and deep understanding of RAUCCHS members’ perspectives on the health of urban First Nations Australians, and their priorities for an Indigenous urban health research agenda, stakeholders working in RAUCCHS member services with longstanding and/or senior roles in the urban ACCHS sector were approached to participate in an interview. Fourteen individuals across the eleven RAUCCHS services were invited by email to participate in an interview. Four participants were unavailable to be interviewed. A total of ten individuals from seven RAUCCHS member services were interviewed. Stakeholder participants were from the urban capitals of Brisbane, Sydney, Adelaide, Perth, Canberra and Darwin, and included four General Practitioners (GP), three Chief Executive Officers (CEO), and three Health Service Managers (HSM). Six stakeholders participants identified as Aboriginal and one as Aboriginal and Torres Strait Islander.

### Data collection

Data was collected from participants using a framework of open-ended questions within a guided topic list designed to explore three broad domains of enquiry: (i) The meaning of Indigenous health; (ii) Key issues for urban Indigenous health; and (iii) Priorities of an urban Indigenous health research agenda. A male non-Indigenous Senior Research Fellow from the UQ Poche Centre for Indigenous Health conducted all interviews. The researcher had a Ph.D. in public health and twenty years’ experience undertaking qualitative research in First Nations communities in Australia. The researcher was unknown to participants at the time of interviews. Interviews employed a research yarning approach whereby participants were initially invited to tell their story of working in an urban ACCHS (Bessarab and Ng’andu, 2010). The interviewer facilitated the discussion in a way that enabled the broad domains of inquiry to emerge from the stories of participants. Interviewer prompts and questions were used to seek additional information or clarify issues. This approach privileged participants’ experiences while conveying meaningful data for research purposes (Bessarab and Ng’andu, 2010). All interviews were conducted virtually, audio recorded and transcribed verbatim. Interviews were 37 to 66 minutes duration. All participants were offered a copy of their interview transcript. One participant took up this option.

### Data analysis

NVivoPro 12 Software was used for data management and analysis. The same researcher that interviewed participants analysed interview data, drawing on key aspects of a well-established thematic analysis approach that uses ‘open’ and ‘axial’ coding (Strauss and Corbin, 1990). Open coding involved reading through interview transcripts to increase familiarity with the material and to prepare ‘theoretical memos’ as analytical reminders for generating ideas and making links between different findings. Axial coding linked and organized open codes that were developed into a coding tree. The coding tree was shown to an Aboriginal female postdoctoral researcher, with clinical, policy and research experience in the Aboriginal community-controlled sector, for her interpretation and feedback. This process assisted with the development of preliminary themes and sub-themes which were discussed with 10 representatives from nine RAUCCHS member services at a face-to-face workshop. Five out of the 10 representatives at the workshop had participated in interviews. Themes were finalized in response to comments and feedback from workshop participants.

## Results

Four main interrelated themes captured ACCHS stakeholders’ perspectives on the key issues in First Nations health and their priorities for a First Nations urban health research agenda in Australia. The terms Aboriginal and non-Aboriginal are used when differentiating between First Nations and non-First Nations Australia participants to be consistent with how participants identified themselves. To preserve participant confidentiality, quotes are attributed to whether they were Aboriginal or non-Aboriginal stakeholders, not their professional role, as this would be too identifiable.

*Theme 1: Effectiveness and efficiency of Indigenous models of primary health care adaptable to changing social and population demographics.*

Participants commonly identified enabling access to comprehensive primary health care for First Nations Australians as their main role in an ACCHS. An emerging challenge to fulfilling this role was the changing social and population demographics of First Nations Australians in urban areas. In particular, participants expressed concern that models of care in urban ACCHS are not responsive enough to community needs in the context of a rapidly growing and changing First Nations urban population.

‘We’re seeing such a huge influx into the urban spaces that is the southeast corner… when I look across some of the data that’s coming out of the Institute [Institute for Urban Indigenous Health], as well as the ABS [Australian Bureau of Statistics]. You look at some of that and you think to yourself there’s no way we can continue to just do the run of the mill expansion plans that we’ve been doing for the last ten years; we’ve got to think of something bigger.’ (P1: Aboriginal stakeholder)

The growth in the number of First Nations Australians accessing urban ACCHS was primarily attributed to an increase in the number of people identifying as First Nations Australian (Carlson, 2016), an increase in the cost of attending mainstream primary care services (largely associated with fewer services being fully funded by government) and the migration of rural and remote First Nations Australians into regional and capital cities for employment and healthcare.

‘And they’re coming to [urban ACCHS] because they can’t access the services they need in rural and remote. Things like dialysis, cancer treatments, etc, but they still need their primary healthcare needs met.’ (P2: Aboriginal stakeholder)

The implementation of novel models of primary health care in urban ACCHS and evaluation of their efficiency and effectiveness was identified by several participants as an important research-related strategy for addressing these issues.

‘There’s been a lot of research in Aboriginal health but it hasn’t actually ended up in any translation into health service delivery. And so I think that we need to now start to shift our focus into developing models of care that can be reported on and analyse those that are actually going to be working for Aboriginal people.’ (P3: non-Aboriginal stakeholder)

*Theme 2: Strengthening care pathways to enable First Nations Australians in urban areas to seamlessly transition between health and social services that they need when they need them.*

Service gaps and barriers to accessing specialist services in urban areas make it difficult for First Nations Australians to transition between levels of health care and the range of health and social services they need when they need them. Collectively, stakeholders identified a limited number of ‘Aboriginal-specific’ drug and alcohol services; a shortage of allied health services; a reduction in the number of health services fully funded by government; long waiting times to access early childhood and developmental services; and disjointed mental health care services. Aboriginal stakeholders, in particular, identified racism as a major barrier to First Nations Australians accessing mainstream services that were available.

‘Racism is a huge barrier to anything. No one wants to go into a place and just feel, they just constantly feel like the “other,” so that to me has a profound effect on how people participate, let their vulnerable parts of themselves show.’ (P4: Aboriginal stakeholder)

Stakeholders’ experiences of the detrimental impact of service gaps and barriers on the health and wellbeing of First Nations Australians were strongly reflected by the high priority they placed on addressing these issues in an urban Indigenous health research agenda.

‘The barriers as to why, researching why urban mob don’t access services. Mainly it’s cultural safety of those services. There might be services available but our mob aren’t accessing it because it’s not culturally safe or they don’t feel culturally safe or they don’t know about it.’ (P5: Aboriginal stakeholder)

*Theme 3: Examine the intersection of identity, racism and determinants of health and their impact on the health and wellbeing of First Nations Australians.*

For Aboriginal stakeholder participants, equally as important as ACCHS enabling access to culturally safe comprehensive health care, was their role in affirming and strengthening Indigenous cultural identity.

‘I think the essence is its Blackness, its ability to stand there, feel like home for people, feel like it’s a comfortable space, you can be vulnerable here, it’s okay, and we are going to hold you and we’re going to care for you, and we’re going to give you the ultimate access to what we’ve got on offer.’ (P1: Aboriginal stakeholder)

Relationships with family, community, and Country were identified as cultural factors that have a profound impact on the cultural identity of First Nations Australians. Aboriginal stakeholders spoke about the struggles urban First Nations Australians have affirming their cultural identity in the face of being perceived as ‘less cultural’, ‘disconnected from Country’, and ‘not a real Blackfella’. They described these stereotypes and misconceptions as pervasive and expressed concern for their damaging impact on the health, social and emotional wellbeing of urban First Nations Australians.

‘Well, the impact is you don’t really belong to anybody and you’re just waiting, I suppose. That builds on your trauma because you’re not connected to any group. Then you’re making up your own culture in a sense, or you’re traumatised because you’re not a part of the community when you’re growing up.’ (P4: Aboriginal stakeholder)

The importance of addressing these factors were reflected in stakeholders’ recommendations for more research on embedding culture in healthcare delivery, developing a multi-level understanding of the interaction of cultural identity with other health determinants; and building a First Nations-led evidence-base around causal pathways.

*Theme 4: Invest in research structures and processes that enable local Indigenous research governance.*

Stakeholders’ experiences were that current research investment in ACCHS is limited to supporting or employing people to do research led by external institutions. They recommended greater investment in structures and processes that enable ACCHS and the communities they serve to have oversight and control over First Nations health research in Australia.

‘If research bodies or universities truly want to collaborate with AMS [Aboriginal Medcial Services] to deliver research projects, which is clearly what they want to do, they need to actually start investing in structures to build research capacity in that AMS.’ (P7: non-Aboriginal stakeholder)

More specifically, stakeholder participants identified a need for significant investment in ‘Indigenous models of research governance’ and ‘Indigenous data sovereignty’. They perceived Indigenous research governance as essential for safely engaging First Nations communities in research and affirming local community and cultural protocols throughout the research process. To enable this, some participants proposed that grants funding First Nations health research include a mandatory component in their budget for building research leadership, capacity and expertise in ACCHS, to ensure they are not merely sites for researchers to access First Nations Australian communities to undertake research. One participant suggested that funding awarded for First Nations health research include Indigenous research governance as a mandatory budget item and that the amount allocated be set as a ‘at a minimum’ percentage (e.g. ≥10%) of the total budget.

First Nations data sovereignty was needed to ensure the collection and use of data was governed by the interests, values, and needs of First Nations Australians. Several participants spoke about the need to align Patient Information Management Systems (PIMS) used in ACCHS with the way care is delivered to enable the collection of data on the level and intricacies of care provided.

‘We need to be able to tell this story about the amount of care that community-controlled health services provide that’s above and beyond what your mainstream general practice does, and why Medicare is probably significantly under-resourcing community-controlled health services.’ (P3: non-Aboriginal stakeholder)

Three participants identified the data and research team at IUIH as a best practice model of First Nations data sovereignty. Key features of the model they highlighted were in-house technical expertise for extracting routinely collected health service data, statisticians for analysing data, and data scientists for developing data visualizations. In describing the overall value and benefit of the model, one Aboriginal participant said:

‘There’s no way the institute (IUIH) would be able to be what they are today if they actually didn’t have control over all that information data that’s being collected that actually demonstrated the evidence to be able to enhance and expand their service delivery across South-East Queensland.’ (P8: Aboriginal stakeholder)

When asked about ways of doing health research with ACCHS, stakeholders commonly talked about ‘co-designed research’. However, interpretations of the meaning of co-design varied depending on participants’ experiences of ACCHS being involved in health research. Participants involved in positive and successful research partnerships between ACCHS and research institutions described co-design as an equitable two-way process in which research institutions and ACCHS relied on each other for research ideas, knowledge, and data.

‘We would access [research institution] as an example. They’re a huge source of research for us. We do a lot of co-designing with them, depending on the programs that we are using. Yes, they have an Indigenous sector in that particular research institute and we do rely on a lot of the data they use just as they rely on the data that they collect from us as well.’ (P4: Aboriginal stakeholder)

Alternatively, participants who expressed frustration at ACCHS being inundated with requests from researchers wanted first and foremost for First Nations research to be designed around ideas coming from First Nations people because, as stated by an Aboriginal participant [P9]: ‘We are experts in telling our story’.

Three Aboriginal participants expressed views that despite growth in the number of First Nations-led research institutes and First Nations researchers, First Nations health research is still being conducted in ways that benefit the careers of researchers more than it does the health of First Nations communities. When they spoke about co-design, they described a process in which researchers were transparent, accountable, followed local protocol, and respected Indigenous ways of knowing, being and doing. These features of co-design articulated by participants are consistent with the key principles of true co-design identified in a critical review of current approaches to co-designing health policy for Aboriginal and Torres Strait Islander peoples (Butler *et al.,* 2025).

‘We did a co-design study…and it took 18 months for communication and rapport-building, until we could even move forward with anything. Even though I was from there I made sure we followed protocol, and protocol means that it’s, as you know, certain steps that you have to follow within that tribe or the tribal system, and I’m not talking about initiation or anything like that, I’m just talking about your respect for Elders, the proper consultation, and that took 18 months to follow protocol, nearly 18 months to follow proper protocol to build what the community, and also the researchers wanted and needed.’ [P9: Aboriginal stakeholder]

## Discussion

The ACCHS stakeholders we spoke to perceived that the health of urban First Nations Australians is underrepresented in First Nations health research in Australia. Their views are supported by multiple reviews of First Nations health research (Eades *et al.,* 2010; Jennings *et al.,* 2021; Kennedy *et al.,* 2022) and recommendations for greater investment in First Nations health research nationally (Stajic *et al.,* 2023) and globally (Mamun *et al.,* 2023). The broad priority areas for UQ Poche Centre’s Indigenous Urban Health Research Agenda identified by stakeholders were evaluating the effectiveness and adaptability of First Nations models of care, strengthening care pathways between ACCHS and specialist services, examining the intersection of cultural identity and racism with determinants of health, and investment in First Nations research governance structures and processes.

There is a lack of research on the effectiveness of models of care in ACCHS (Harfield *et al.,* 2018), despite these services being the main provider of primary health care (AIHW, 2023) to a rapidly growing First Nations Australian population (Australian Bureau of Statistics, 2021). Growth is highest in urban areas and is linked to an increase in migration from rural and remote areas and changes in how individuals report their Aboriginal and/or Torres Strait Islander status (Andrews and Markham, 2022). As highlighted by stakeholders, the impact of rapid population growth on access to health care for the urban First Nations Australian population is compounded by the lack of mainstream services adequately serving their needs (Australian Institute of Health and Welfare, 2023). Moreover, stakeholders’ views that ACCHSs in urban areas are under resourced to meet the growing demand for their services is supported by qualitative (Clifford-Motopi *et al.,* 2024) and empirical evidence (Taylor *et al.,* 2021). In response, some urban ACCHSs are adapting their models and systems of health care to better meet the healthcare needs of a rapidly growing First Nations Australian population (Butler *et al.,* 2022). Two recent examples are IUIH’s implementation and evaluation of an adapted model of a Patient Centred Medical Home (PCMH) (Butler *et al.,* 2022) and redesigned system of birthing and maternal services (Kildea *et al.,* 2018). Rigorous evaluations of these models and systems of care were possible because of structures and processes that enabled ACCHS to have oversight and control over research, thereby ensuring that Indigenous worldviews, knowledge, realities and terms of reference were embedded throughout all stages of the research process (Kildea *et al.,* 2018; Butler *et al.,* 2022). The outcomes of these evaluations demonstrate that First Nations-led research is crucial for building the evidence base of models and systems of health care likely to translate into significant improvements in health care access, utilization (Davy *et al.,* 2016; Kildea *et al.,* 2019; Mathew *et al.,* 2023) and health outcomes (Kildea *et al.,* 2019) for urban First Nations Australians.

The barriers First Nations Australians face accessing specialist services identified by ACCHS stakeholders are well documented (Davey, 2014; Nolan-Isles *et al.,* 2021; Australian Institute of Health and Welfare, 2023). As is their impact on the rates of First Nations Australians accessing specialist services. In 2017–18, the Medicare claim rate for specialist MBS services among First Nations Australians in major cities was 860 per 1000 population compared to 1145 per 1000 for non-Indigenous Australians (Australian Institute of Health and Welfare, 2023). This was despite First Nations Australians having greater health needs. There is a decline in MBS service usage with remoteness, especially for specialist services (Australian Institute of Health and Welfare, 2023). ACCHS stakeholders’ views that this gradient results in more remote-living First Nations Australians moving to larger urban centres where more health services are located, increasing the demand for Indigenous primary health care and specialist care in these areas, has been identfied as one factor contributing to the urbanization of Indigenous people (Mamun *et al.,* 2023). The high priority stakeholders placed on research aimed at strengthening care pathways between different types of health services in urban areas, not only reflects the multiple barriers preventing First Nations Australians from accessing specialist services when they need them, but also the lack of evaluations of programs designed to address them (Ware, 2013).

Racism is a public health issue and a prominent and persistent barrier to First Nations Australians accessing healthcare (Gatwiri *et al.,* 2021; Watego *et al.,* 2021). Aboriginal stakeholders’ narratives of First Nations Australians feeling ‘othered’ and ‘culturally unsafe’ in mainstream health care reflects negative racially based treatment more broadly (Larson *et al.,* 2007; Watego *et al.* , 2021). Furthermore, their experiences that urban First Nations Australians are not considered ‘authentically’ Indigenous, due to their greater heterogeneity relative to their remote-living counterparts, and the harmful affect this can have on their cultural identity, has been written about previously (Fredericks Bronwyn, 2013; Brand *et al.,* 2016). Brand *et al.*, 2016 contend that such notions of Indigeneity causes ‘Indigenous invisibility’ in urban areas, compromising service delivery to urban First Nations Australians (Brand *et al.,* 2016). Moreover, racism is foundational to the production of health inequities (Watego *et al.,* 2021) and associated with negative health outcomes for First Nations Australians (Kairuz *et al.,* 2021). Despite the persistence of racism in health care and its detrimental impact on health outcomes, there is a lack of evidence on how best to reduce racism in these settings. Importantly, the Lowitja Institute advocates that First Nations health research have a stronger focus on addressing racism in health care and understanding how race operates in the production of health inequities. To enable this, Lowitja recommend more targeted investment in race and racism research by health research funding bodies (Watego *et al.,* 2021).

Strengthening the capacity of ACCHS to lead research by greater investment in Indigenous models of research governance and data sovereignty were overarching priorities among stakeholders. A recent study examining ethics guidelines use and Indigenous governance and participation in Aboriginal and Torres Strait Islander health research among researchers found that Indigenous governance and participation was inadequate at each stage of research (Burchill *et al.,* 2023). Birchall et al., conclude: ‘Re-orientation and investment are needed to give control of the framing, design, and conduct of Indigenous health research to Indigenous people’ [33 p. 89]. Globally, First Nations leaders and organizations are calling for structural changes that enable greater self-determination, leadership, and control of health services (Nations, 2007). Indigenous data sovereignty reaffirms the rights of Indigenous Peoples to control data about their peoples, lands, and resources (Maiam nayri Wingara Indigenous Data Sovereignty Collective, 2024). Indigenous research governance enacts those rights through mechanisms grounded in Indigenous rights and interests that promote Indigenous values and equity (Maiam nayri Wingara Indigenous Data Sovereignty Collective, 2024). Adequate investment in Indigenous models of research governance transfers decision making to First Nations Australians on health priorities and the types of research needed, facilitating research that is relevant, ethical, and capable of delivering transformative health outcomes (Bond *et al.,* 2016; Hickey *et al.,* 2018; Turner *et al.,* 2019; Butler *et al.,* 2022). Paradoxically, guidelines for advancing Indigenous governance and participation in research, developed by the National Health & Medical Research Council (NH&MRC) i.e., the primary source of funding for Indigenous health research in Australia, are not mandatory (National Health and Medical Research Council, 2018a; 2018b). Nor is it a requirement that researchers applying to the NH&RMC for Indigenous health research funding allocate a set minimum proportion of their budget to investing in these actions. It is therefore not surprising that the extent to which these guidelines are implemented by research institutions and researchers varies considerably (Burchill *et al.,* 2023). Proper Indigenous governance and participation in research requires a considerable amount of time and financial investment to develop partnerships between researchers and communities that embed Indigenous ways of knowing, being and doing throughout all stages of the research process (Bond *et al.,* 2016; Harfield *et al.,* 2020). For ACCHS stakeholders, proper Indigenous-led research partnerships means co-designing studies around the priorities, protocols, and timelines of First Nations Australians communities. The Research Alliance of Urban Community-Controlled Health Services (RAUCCHS) (Stajic *et al.,* 2023) and Research Alliance for Urban Goori Health (RAUGH) are two examples of such partnerships. (RAUGH. The Research Alliance for Goori Health).

The research priorities identified by RAUCCHS members will be a main focus of the research themes articulated in the UQ Poche Centre Indigenous urban health research agenda. This will ensure that the research needs of urban ACCHS and the communities they serve are prioritized in UQ Poche Centre’s Indigenous health research partnerships, grants, and publications.

### Limitations

The results of our study are limited as they only reflect the views of RAUCCHS members. Similar research that elicits views from stakeholders working in other urban First Nations health services in Australia would expand our findings. In targeting urban ACCHS in capital cities we were unable to recruit participants from Melbourne and Hobart for interviews. The perspectives of ACCHS stakeholders from these cities may have differed from those who participated in the study. Two stakeholders from the RAUCCHS service in Melbourne, however, contributed their perspectives by participating in the workshop at the national RAUCCHS meeting.

## Conclusion

The research priorities of urban ACCHS were to strengthen First Nations models and systems of care and examine the impact of the intersection of cultural identity and racism with determinants of health. Addressing these priority areas requires considerably more effort and investment in First Nations models of research governance and data sovereignty, to ensure that control and decision-making is rightfully in the hands of First Nations peoples. Accordingly, a UQ Poche Centre Indigenous Urban Health Research Agenda will enable research to be driven by communities and conducted by a First Nations-led research centre. In doing so, the agenda calls on governments and research funding bodies to reimagine understandings of First Nations health research in Australia and to provide greater policy focus and funding allocation to urban First Nations health research.

## Supporting information

Clifford-Motopi et al. supplementary material Clifford-Motopi et al. supplementary material

## Supplementary material

The supplementary material for this article can be found at https://doi.org/10.1017/S1463423625100807.
